# A Stepwise Approach Using Metastatic Lymph Node Ratio-Combined Thyroglobulin for Customization of [^18^F]FDG-PET/CT Indication to Detect Persistent Disease in Patients with Papillary Thyroid Cancer

**DOI:** 10.3390/diagnostics11050836

**Published:** 2021-05-06

**Authors:** Hong Hua Piao, Subin Jeon, Su Woong Yoo, Young Jae Ryu, Dong-Yeon Kim, Ayoung Pyo, Hee-Seung Bom, Jung-Joon Min, Seong Young Kwon

**Affiliations:** 1 Department of Nuclear Medicine, Chonnam National University Hwasun Hospital, Hwasun-gun 58128, Korea; honghua0307@hanmail.net (H.H.P.); subin4255@naver.com (S.J.); yoosw.md@gmail.com (S.W.Y.); payjal@naver.com (A.P.); hsbom@jnu.ac.kr (H.-S.B.); jjmin@jnu.ac.kr (J.-J.M.); 2 Department of Surgery, Chonnam National University Medical School and Hwasun Hospital, Hwasun-gun 58128, Korea; brandon-surgery@hotmail.com; 3 College of Pharmacy and Research Institute of Pharmaceutical Science, Gyeongsang National University, Jinju 52828, Korea; blueburr@gmail.com; 4 Department of Nuclear Medicine, Chonnam National University Medical School, Hwasun-gun 58128, Korea

**Keywords:** papillary thyroid cancer, thyroglobulin, lymph node ratio, persistent disease, [^18^F]FDG PET/CT

## Abstract

We investigated whether an indication for [^18^F]FDG-PET/CT to detect FDG-avid persistent disease (PD) could be identified precisely using the extent of metastatic lymph nodes (MLNs) and serum thyroglobulin (Tg) in papillary thyroid cancer (PTC) patients. This retrospective study included 429 PTC patients who underwent surgery and radioactive iodine (RAI) therapy. [^18^F]FDG-PET/CT and serum Tg were evaluated just before RAI therapy. The MLN ratio (LNR) was defined as the ratio of the number of MLNs to the number of removed LNs. To derive the LNR-combined criteria, different Tg cut-off values for identifying the PET/CT-indicated group for PD detection were applied individually to subgroups initially classified based on LNR cut-off values. The cut-off values for serum Tg, the number of MLNs, and LNR for a PET/CT indication were 6.0 ng/mL, 5, and 0.51, respectively. Compared to a single parameter (serum Tg, total number of MLNs, and LNR), the LNR-combined criteria showed significantly superior diagnostic performance in detecting FDG-avid PD (*p* < 0.001). The diagnostic performance of PET/CT in detecting FDG-avid PD was significantly improved when the PET/CT-indicated group was identified through the LNR-combined criteria in a stepwise manner; this can contribute to a customized PET/CT indication in PTC patients.

## 1. Introduction

Well-differentiated thyroid carcinoma (DTC) is the most common thyroid malignancy and generally has an excellent prognosis and a low mortality rate [1,2]. Despite its good prognosis, 3–10% patients could have persistent or recurrent disease (PRD) within the first decade after surgery and radioactive iodine (RAI) therapy [3]. Compared to recurrent disease (RD), persistent disease (PD) has different characteristics and requires a different management approach; however, the corresponding disease definitions and timelines have not been defined clearly. In patients with PD, therapeutic response to RAI therapy is often poor, and life expectancy could be significantly reduced [4]. Bates et al. [5] reported that multiple reoperations in patients with DTC were performed for the management of PD and not for the management of RD. Therefore, early detection of PD after surgery followed by RAI therapy is very important for formulating a disease management plan for patients with DTC.

2-deoxy-2-[^18^F]fluoro-D-glucose ([^18^F]FDG) positron emission tomography/computed tomography (PET/CT) is a useful imaging modality for detecting PD after surgery [6]. It is necessary to identify FDG-avid PD by elaborating a PET/CT indication because positive FDG uptake lesions have been suggested to have poor response to RAI therapy and a worse prognosis [7,8]. In previous studies, the diagnostic performance of PET/CT was associated with serum thyroglobulin (Tg) levels [9,10]. However, serum Tg levels could be elevated not only in patients with PRD but also in those with normal remnant thyroid tissues. Furthermore, serum Tg levels may be influenced by several factors such as the assay method, thyroid-stimulating hormone (TSH) level, and endogenous anti-Tg autoantibodies (TgAbs), resulting in false-negative results [11]. Therefore, other parameters to complement serum Tg should be addressed to more precisely establish an [^18^F]FDG-PET/CT indication.

Neck lymph node (LN) metastases could be one of the most common sites of PRD in patients with DTC [12]. Increasing evidence suggests that the extent of metastatic LNs (MLNs) could play an important role in the evaluation of prognosis and further management in patients with DTC [13]. The extent of MLNs could be evaluated using the total number of MLNs or MLN ratio (LNR), which is defined as the ratio of the number of MLNs to the number of removed LNs. Several studies have showed that LNR could be an important prognostic factor in patients with DTC [14,15,16]. However, there are limited studies on the application of the extent of metastatic LNs to detect FDG-avid PD in patients with DTC.

In this study, we investigated whether an indication for [^18^F]FDG-PET/CT to detect FDG-avid PD could be derived more precisely using the extent of MLNs (number of MLNs or LNR) and serum Tg, and whether the diagnostic performance of PET/CT could be improved by combining the extent of MLNs and serum Tg in patients with papillary thyroid cancer (PTC).

## 2. Materials and Methods

### 2.1. Patients

We initially reviewed data on 623 patients with PTC who underwent [^18^F]FDG-PET/CT 4–12 days before RAI therapy following total thyroidectomy (with central and/or lateral neck dissection) between January 2008 and December 2014. The exclusion criteria were as follows: (1) other primary cancer at the time of diagnosis (*n* = 34), (2) distant metastasis detected before or after RAI therapy (*n* = 5), (3) serum TgAb level ≥100 IU/mL (*n* = 72) or TSH level <30 mIU/L (*n* = 8) before RAI therapy, and (4) insufficient data in medical records (*n* = 75). Finally, 429 patients were enrolled in this study. The enrolled patients had discontinued levothyroxine for 4 weeks and had received supplementary triiodothyronine for the first 2 weeks prior to RAI therapy for increasing the TSH level. In addition, patients were placed on a low-iodine diet for 2 weeks prior to RAI therapy.

This retrospective study was approved by the institutional review board of our hospital, and the requirement for informed consent was waived.

### 2.2. Assays

Serum Tg levels were evaluated just before RAI therapy (D0Tg); Tg levels were measured by immunoradiometric assay (IRMA) (RIA Tg-plus, BRAHMS GmbH, Hennigsdorf, Germany) with a lower detection limit of 0.2 ng/mL. TgAb and TSH levels were also evaluated just before RAI therapy. Serum TgAb levels were measured by radioimmunoassay (RIA anti-Tgn, BRAHMS GmbH, Hennigsdorf, Germany), with a lower detection limit of 20 U/mL. Serum TSH levels were determined by IRMA (TSH-CTK-3, DiaSorin, Saluggia, Italy), with a lower detection limit of 0.07 µIU/mL. In all patients, serum TSH levels were >30 µIU/mL prior to RAI administration.

### 2.3. Image Acquisition

PET/CT images were obtained using the Discovery ST PET/CT system (GE Medical Systems, Milwaukee, WI, USA). Briefly, all patients fasted for 6 h before intravenous injection of [^18^F]FDG. [^18^F]FDG (5.55 MBq/kg body weight) was administered to each patient intravenously 50 min prior to imaging. Patient orientation was HFS. For attenuation correction, we acquired a non-contrast-enhanced low-dose CT scan. Then, PET images were acquired for 150 s per bed position. Data were reconstructed using ordered subset expectation maximization reconstruction (128 × 128 matrix, 3.27-mm slice thickness, 3.9 × 3.9 mm pixel size, subsets: 16 or 21, iterations: 2). The average transverse spatial resolution at 1, 10, and 20 cm off axis was 6.68, 7.72, and 8.13 mm in 3D, respectively. Contrast medium was not used in the PET/CT study to prevent effects on RAI uptake in remnant thyroid tissues. PET/CT protocol was managed in accordance with the guidelines set by our society of nuclear medicine.

An iodine-whole-body scan (IWBS) was performed using two variable angle dual-head gamma cameras (Millennium VG and Infinia Hawkeye 4; GE Medical Systems, Milwaukee, WI, USA). IWBS images were obtained 2 or 7 days after the administration of a high dose of ^131^I for therapeutic purposes.

### 2.4. Study Design

FDG-avid PD was defined when the lesions satisfied the following criteria: (1) discernible focal FDG uptake compared with the surrounding normal tissue on PET/CT and (2) the same FDG uptake lesion, pathologically confirmed as a malignant or metastatic lesion. The PET/CT-indicated group was defined as patients with potential benefits from [^18^F]FDG-PET/CT to detect FDG-avid PD.

**Figure 1:**
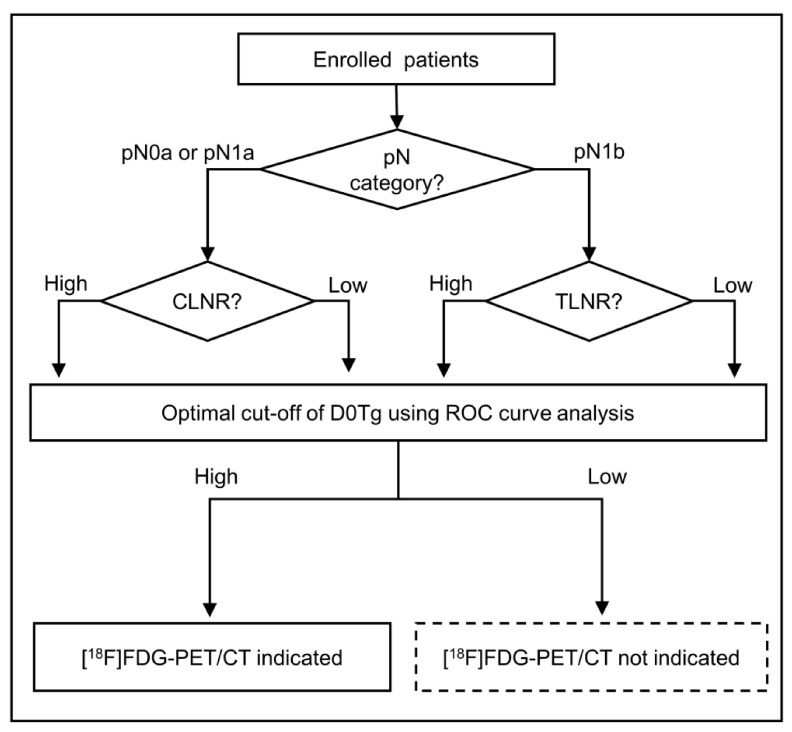
Diagnostic flow-chart for the identification of [^18^F]FDG-PET/CT indication to detect FDG-avid persistent disease using metastatic lymph nodes ratio (LNR) and serum thyroglobulin (D0Tg). LNR was defined as the number of metastatic lymph nodes (MLNs) divided by the number of removed LNs. The optimal cut-off values of LNR and serum Tg were determined using the receiver operating characteristic curve analysis in each steps. (CLNR, central metastatic lymph nodes ratio; D0Tg, serum level of thyroglobulin (ng/mL) checked immediately before radioactive iodine therapy; TLNR, total metastatic lymph nodes ratio).

LNR was defined as the ratio of the number of MLNs to the number of removed LNs. We also tried to exclude patients in whom less than three LNs were retrieved during surgery to avoid falsely exaggerating the LNR [16]; however, none of the patients fulfilled this exclusion criterion. After evaluating clinicopathological factors to identify FDG-avid PD using receiver operating characteristic (ROC) curve analysis, we determined the optimal cut-off value of each parameter (such as the number of MLN, LNR, or D0Tg level) to identify the PET/CT-indicated group. We further evaluated whether a PET/CT indication could be identified precisely through a stepwise approach based on the combination of LNR and D0Tg (Figure 1). Initially, all patients were classified into two groups according to the pathological N category—pN0a or pN1a and pN1b. The two groups were further divided into four subgroups based on LNR cut-off values determined by ROC curve analysis. Central LNR (CLNR) or total LNR (TLNR) was applied according to the pathological N category. Patients with a high D0Tg level (higher than the cut-off value) were allocated to the PET/CT-indicated group if an optimal cut-off of D0Tg by ROC curve analysis was available for each subgroup. If a cut-off value of D0Tg was not available, the PET/CT-indicated group was evaluated based on the cut-off value of CLNR or TLNR from the prior step. We investigated whether the diagnostic performance of [^18^F]FDG-PET/CT to detect FDG-avid PD was improved when the PET/CT-indicated group was identified using the LNR-combined criteria compared that using a single parameter. We tried to apply the number of MLN-combined criteria in a similar way, but the diagnostic performance of [^18^F]FDG-PET/CT was not improved compared to that using the LNR-combined criteria.

In addition, we further evaluated whether iodine uptake patterns on IWBS were different according to the presence of FDG-avid PD in the PET/CT-indicated group identified based on the LNR-combined criteria. Iodine uptake positivity was defined in patients who had iodine uptakes in both the midline and thyroidectomy bed of the anterior neck on IWBS. We compared the proportion of iodine uptake-positive patients between the two groups with or without FDG-avid PD.

All [^18^F]FDG-PET/CT and IWBS images were reviewed independently by two experienced nuclear medicine physicians.

### 2.5. Statistical Analyses

Descriptive quantitative data are presented as means ± standard deviations or median (ranges). Qualitative data are expressed as percentages. The differences in variables were evaluated using Student’s t test or the chi-square test for continuous and categorical variables, respectively. Multivariate logistic regression analysis was performed to evaluate clinicopathological factors identifying FDG-avid PD. McNemar’s test was used to compare the diagnostic performance of [^18^F]FDG-PET/CT for FDG-avid PD detection through the identification of a PET/CT indication using the LNR-combined criteria and that using a single parameter. ROC curve analysis was performed to identify the optimal cut-off values of parameters in each step. *p*-values < 0.05 were considered statistically significant. All statistical analyses were performed using IBM SPSS for Windows^®^, version 21.0 (IBM Corp., Armonk, NY, USA).

## 3. Results

### 3.1. Patient Characteristics

The clinicopathological characteristics of the enrolled patients are summarized in Table 1. The study group consisted of 94 (21.9%) males and 335 (78.1%) females. The patients were 17–83 years old (mean, 46.6 years old) at the time of RAI therapy. The histological DTC subtype was papillary in all patients. The mean diameter of the primary tumor was 1.2 ± 0.9 cm (range, 0.2–6.0 cm), and 232 (54.1%) of 429 patients had a tumor size ≥ 1.0 cm. Unilateral tumors were more frequently detected than bilateral tumors (72.5% vs. 27.5%). Extra-thyroidal extension (ETE) was not detected in 264 (61.5%) patients. The most prevalent category of primary tumors was T1a (*n* = 209, 48.7%). LN metastasis was found in 93.3% of patients, and pN1a category tumors (*n* = 304, 70.9%) were more prevalent than pN1b category tumors (*n* = 96, 22.4%). The median values of the number of MLNs, LNR, and D0Tg were 2 (range, 0–41), 0.4 ± 0.3 (range, 0.0–1.0), and 8.1 ± 38.2 ng/mL (0.0–500.0 ng/mL), respectively.

### 3.2. Clinicopathological Factors Related to FDG-avid PD on [^18^F]FDG-PET/CT

Thirty-two (7.5%) patients had pathologically confirmed FDG-avid PD. When the maximum standardized uptake value (SUVmax) was evaluated in these patients, the mean value of SUVmax was 5.6 ± 5.7 (range, 1.9–26.7). We investigated the parameters that were significant for detecting FDG-avid PD on PET/CT after surgery (Table 2). In univariate analysis, being male (*p* = 0.027), primary tumor size ≥ 1.0 cm (*p* = 0.001), the presence of gross ETE (*p* = 0.001), pT ≥ 3b (*p* < 0.001), pN1b (*p* < 0.001), high number of MLNs (*p* < 0.001), high LNR (*p* = 0.004), and high serum D0Tg levels (*p* < 0.001) were significantly associated with the detection of FDG-avid PD. Multivariate logistic regression analysis showed that only D0Tg was significantly associated with the detection of FDG-avid PD (odds ratio 1.043; 95% confidence interval 1.025–1.062; *p* < 0.001).

### 3.3. Identification of the PET/CT-Indicated Group for Detecting FDG-avid PD Using the Combined Criteria Including MLN Ratio and Serum Tg

**Figure 2:**
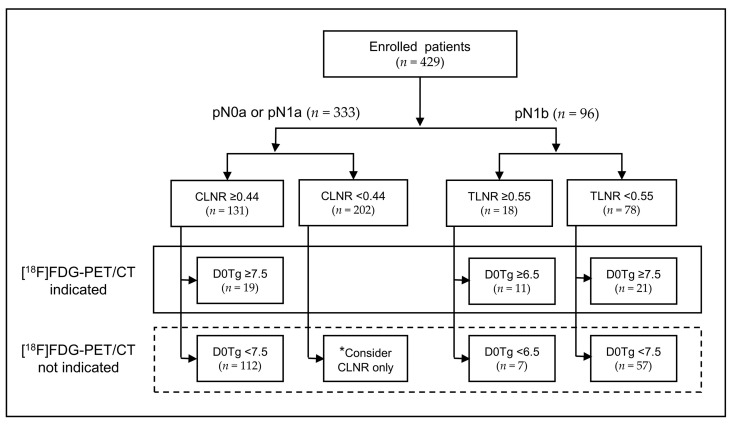
Distribution of patients according to the diagnostic criteria using the metastatic lymph nodes ratio (LNR) and the serum level of thyroglobulin obtained immediately before RAI therapy (D0Tg). * All patients with central LNR <0.44 were classified into the group with no indication for [^18^F]FDG-PET/CT because the optimal cut-off value of D0Tg was not available. (CNLR, central metastatic lymph nodes ratio; D0Tg, serum level of thyroglobulin (ng/mL) obtained immediately before RAI therapy; TLNR, total metastatic lymph nodes ratio).

Based on a step-wise approach including a combination of LNR and D0Tg (Figure 1), the distribution of the patient population with reference to the identification of an [^18^F]FDG-PET/CT indication is shown in Figure 2. According to ROC curve analysis, the optimal cut-off value of LNR for the identification of a PET/CT indication was different according to pN category as follows: CLNR at 0.44 in pN0a or pN1a and TLNR at 0.55 in pN1b. The optimal cut-off value of D0Tg was also determined in four subgroups based on CLNR or TLNR: high CLNR (≥0.44, *n* = 131), low CLNR (<0.44, *n* = 202), high TLNR (≥0.55, *n* = 18), and low TLNR (<0.55, *n* = 78). In the pN1b group, the cut-off values of D0Tg were slightly different between the two subgroups (6.5 ng/mL in the high TLNR and 7.5 ng/mL in the low TLNR subgroups). In the pN1a group, the cut-off value of D0Tg was 7.5 ng/mL in the high CLNR subgroup. However, the cut-off value of D0Tg was not determined in the low CLNR subgroup, which was allocated to the PET/CT not indicated group. Finally, 51 (11.9%) patients with high D0Tg levels in each subgroup were categorized into the PET/CT-indicated group.

### 3.4. Comparison of the Diagnostic Performance of [^18^F]FDG-PET/CT for Detecting PD

The diagnostic performance of PET/CT for the detection of FDG-avid PD for each [^18^F]FDG-PET/CT indication using the LNR-combined criteria and a single parameter was compared (Table 3). The optimal cut-off values of D0Tg, the number of MLNs, and LNR for detecting FDG-avid PD on PET/CT were 6.0 ng/mL, 5, and 0.51, respectively. The sensitivity, specificity, positive predictive value (PPV), negative predictive value (NPV), and accuracy were as follows: D0Tg (cut-off value, 6.0 ng/mL): 84.4%, 89.2%, 38.6%, 98.6%, and 88.8%, respectively; the number of MLNs (cut-off value, 5): 53.1%, 81.1%, 18.5%, 95.6%, and 79.0%, respectively; and LNR (cut-off value, 0.51): 59.4%, 74.6%, 15.8%, 95.8%, and 73.4%, respectively. In contrast, the sensitivity, specificity, PPV, NPV, and accuracy using the LNR-combined criteria were 78.1%, 93.5%, 49.0%, 98.2%, and 92.3%, respectively, which indicated significantly better diagnostic performance than that using a single parameter (*p* <0.001, respectively).

### 3.5. Distributions of Iodine Uptake Patterns According to the Presence of FDG-avid PD

**Figure 3:**
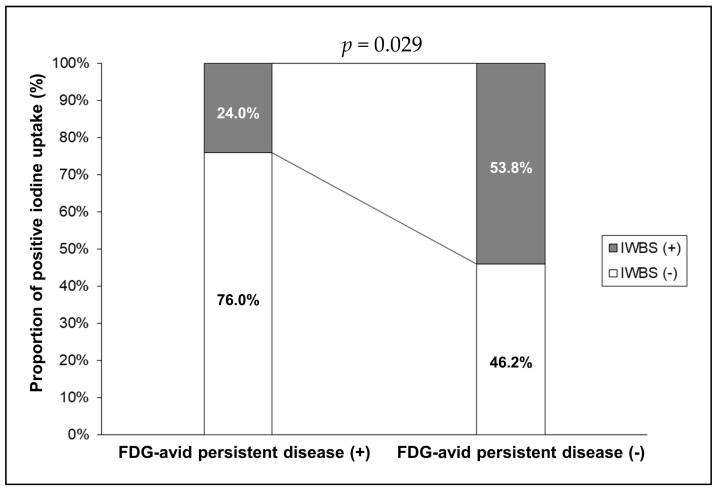
Comparison of iodine uptake positivity on a post-therapeutic iodine whole-body scan (IWBS) according to the presence of FDG-avid persistent disease (PD). Among the 51 patients for whom a [^18^F]FDG-PET/CT was indicated based on the metastatic lymph node ratio-combined criteria, iodine uptake positivity was defined in those with iodine uptakes in the midline of the anterior neck and in the thyroidectomy bed on IWBS. The proportion of iodine uptake-positive cases was significantly higher in the group without FDG-avid PD (14 of 26 [53.8%] patients) than in the group with FDG-avid PD (6 of 25 [24.0%] patients) (*p* = 0.029).

We evaluated whether iodine uptake patterns on IWBS were different according to the presence of FDG-avid PD in 51 patients in the PET/CT-indicated group based on the LNR-combined criteria (Figure 3). The proportion of patients with a positive iodine uptake was significantly higher among those without FDG-avid PD than among those with FDG-avid PD (53.8% vs. 24.0%, *p* = 0.029).

**Figure 4:**
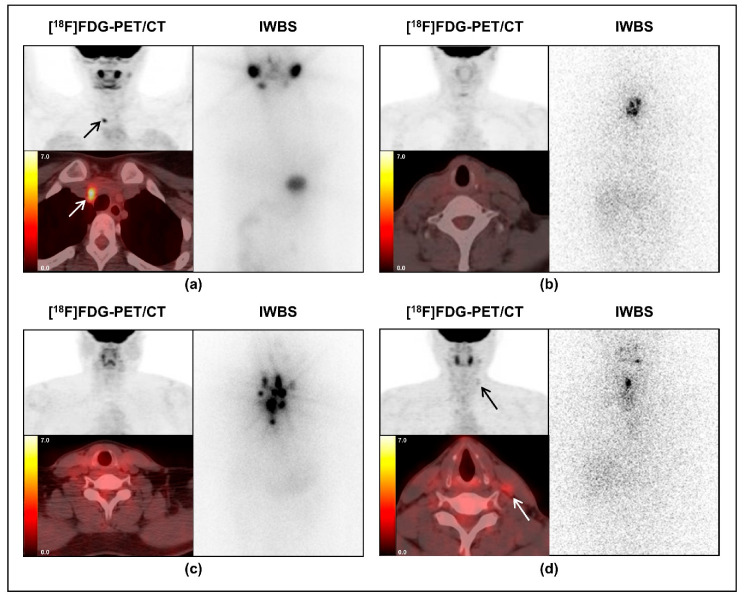
Differences in the diagnostic performance of PET/CT for detecting FDG-avid persistent disease (PD) based on the metastatic lymph node ratio (LNR)-combined criteria, and comparison with post-therapeutic iodine-whole-body scan (IWBS) in patients with papillary thyroid cancer (PTC). (**a**) An 18-year-old woman with a high central LNR (CLNR) of 1.00 was indicated for [^18^F]FDG-PET/CT because the serum thyroglobulin level immediately before RAI therapy (D0Tg, 16.0 ng/mL) was above the cut-off value (≥7.5 ng/mL). This patient had focal FDG uptake at the right neck level VI (black and white arrows, SUVmax: 4.9), which was pathologically revealed as PD. There was no significant iodine uptake in the thyroidectomy bed or in the midline neck area on IWBS. (**b**) A 46-year-old woman with a low CLNR of 0.16, which was less than the cut-off value (0.44), was not indicated for [^18^F]FDG-PET/CT despite of a high D0Tg level (10.0 ng/mL). This patient had no FDG-avid PD on PET/CT; however, several iodine uptake areas were observed in the anterior neck on IWBS. (**c**) A 40-year-old man with a high CLNR of 1.00 was initially indicated for [^18^F]FDG-PET/CT because of a high serum D0Tg level (21.0 ng/mL) according to the criteria. However, there was no FDG-avid PD on PET/CT. Instead, there were multiple iodine uptake areas in the anterior neck on IWBS. (**d**) A 29-year-old man with a high CLNR of 0.53 had FDG-avid PD at the left neck level III (black and white arrows, SUVmax: 2.9), although he was not indicated for [^18^F]FDG-PET/CT because of a low D0Tg level (1.0 ng/mL). An IWBS showed iodine uptake only in the midline of the anterior neck. (IWBS, iodine-whole-body scan).

The detection rate of FDG-avid PD, according to the LNR-combined criteria, and the comparison of [^18^F]FDG-PET/CT with IWBS in patients with PTC are shown in Figure 4.

## 4. Discussion

In this study, a PET/CT indication for the detection of FDG-avid PD could be precisely identified using the combined criteria, including LNR and D0Tg, in patients with PTC. The diagnostic ability of the combined criteria was superior to that using a single parameter. In this study, cut-off values of LNR or D0Tg were determined differently according to the pN category or the LNR of the prior step, respectively. Iodine uptake patterns on IWBS were also different according to the presence of FDG-avid PD in 51 patients in the [^18^F]FDG-PET/CT-indicated group based on the LNR-combined criteria.

Several issues need to be considered while customizing [^18^F]FDG-PET/CT indication in patients with DTC [17,18]. Bates et al. [5] reported that a significant number of patients with DTC who have undergone reoperation due to RD should be reclassified as having PD. Furthermore, the therapeutic response to RAI therapy is often poor in patients with PD. This implies that disease management should be tailored in such patients, such as performing reoperation instead of repetitive RAI therapy or regular follow-up. Compared to RD, early detection of PD could be the main clinical role of [^18^F]FDG-PET/CT in patients with DTC. To identify an [^18^F]FDG-PET/CT indication for PD detection, serum Tg level before RAI therapy is a well-established parameter [19,20]. In a recent study, the diagnostic performance of PET/CT for PRD detection was significantly improved by Tg doubling time with an optimal threshold of 2.5 years in DTC patients with negative IWBS findings [21]. However, serum Tg levels could be influenced by several factors such as serum TgAb level and TSH stimulation methods [22]. Therefore, other parameters to complement serum Tg should be addressed to establish a PET/CT indication more precisely.

Several studies have reported that the extent of MLNs evaluated using the number of MLNs and LNR was associated with prognosis in patients with DTC [15,23]. Lee et al. [24] reported that PTC patients with ≥6 MLNs were at a 3.7-fold higher risk of recurrence than patients with <6 MLNs. Consequently, the number of MLN was added to the revised ATA guidelines as a factor that distinguishes between low and intermediate risk of recurrence [13]. Schneider et al. [16] reported that an LNR of 0.42 was an independent prognostic factor. A large number of MLNs or a high LNR could increase the risk of hidden metastatic LNs not dissected during surgery; the number of MLNs could be a potential parameter to identify a PET/CT indication for PD detection. In our study, the number of MLNs and LNR values were significantly higher in patients with FDG-avid PD than in those without FDG-avid PD (Table 2) in univariate analysis. However, the diagnostic value of using the number of MLNs or LNR was not high for the detection of FDG-avid PD compared to that of using D0Tg (Table 3). We applied a combined parameter using the extent of MLNs and serum Tg to optimize the [^18^F]FDG-PET/CT indication. As diagnostic performance was not improved with the number of MLN-combined criteria, we applied the LNR-combined criteria to identify a PET/CT indication. We then compared the diagnostic performance of the LNR-combined criteria and a single parameter.

The optimal cut-off value of D0Tg or LNR for a PET/CT indication was different according to subgroups. In the pN1b group, the cut-off value of D0Tg was 6.5 ng/mL in the high TLNR (≥0.55) and 7.5 ng/mL in the low TLNR (<0.55) subgroups. In the pN0a or pN1a groups, the cut-off value of D0Tg was 7.5 ng/mL in the high CLNR subgroup (≥0.44). However, the D0Tg cut-off value was not determined in the low CLNR subgroup (<0.44; Figure 2). These results suggest that when the influence of a risk factor such as LNR increases, the cut-off value of the other parameter (D0Tg) should be applied at a lower range. In other words, the cut-off criteria for parameters such as serum Tg should be applied differently in consideration of other factors.

Although the diagnostic performance of PET/CT for detecting FDG-avid PD improved when the LNR-combined criteria were applied, the PPV was relatively low (49.0%), suggesting that 51.0% patients with a PET/CT indication did not have FDG-avid PD, despite having a high D0Tg level. Furthermore, the PPV differed for each subgroup; 7 of 19 (36.8%) in the high CLNR subgroup, 9 of 11 (81.8%) in the high TLNR subgroup, and 9 of 21 (42.9%) in the low TLNR subgroup (Figure 2). If patients with a PET/CT indication did not have FDG-avid PD, the observed D0Tg may be attributed to the normal remnant thyroid tissue. Previously, RAI uptake in the midline of the anterior neck was related to a relatively large amount of remnant thyroid tissue [25,26]. Thus, we analyzed the differences in iodine uptake patterns on IWBS according to the presence of FDG-avid PD in 51 patients in the [^18^F]FDG PET/CT-indicated group based on the LNR-combined criteria (Figure 3). The proportion of patients with a positive iodine uptake in both the midline and thyroidectomy bed of the anterior neck was significantly higher among patients without FDG-avid PD than in those with FDG-avid PD. However, the diagnostic performance did not improve when the iodine uptake pattern on IWBS was applied to the diagnostic flow-chart (Figure 1).

The incidence of DTC in women is usually higher than that in men. On the other hand, men with DTC have a worse prognosis than women [27,28]. Our study showed that males were significantly associated with the detection of FDG-avid PD in univariate analysis (*p* = 0.027, Table 2). This result implicated that the difference in therapeutic response or prognosis by sex might be related to the prevalence of FDG-avid PD. Further studies are needed to precisely establish the subgroup in which the iodine uptake pattern or sex could be applied to optimize the PET/CT indication.

This study has several limitations. First, because of the retrospective study design, selection bias was inevitable. Second, LNR could be affected by the surgical extents and methods, especially in patients with pN1b category tumors. Although we tried to exclude patients in whom less than three LNs were retrieved during surgery to avoid falsely exaggerating the LNR with reference to previous studies, further studies are needed to measure the extent of MLNs more precisely. Third, we also excluded patients with high TgAb levels to derive sophisticated criteria based on D0Tg and LNR for the identification of a PET/CT indication. To identify PET/CT indications that can be applied to a wider array of patients with DTC, it is necessary to develop a stepwise diagnostic flow including TgAb.

## 5. Conclusions

The diagnostic performance of PET/CT in detecting FDG-avid PD was significantly improved when the PET/CT-indicated group was identified through a combined approach using LNR and serum Tg in a stepwise manner compared to an approach using a single conventional parameter. The study results could help identify an [^18^F]FDG-PET/CT indication for the early detection of PD and aid in the formulation of an appropriate management plan for patients with PTC.

## Figures and Tables

**Table 1:** Patient characteristics (*n* = 429).

Parameters	Values
Age (years)	
Mean ± SD ^1^ (range)	46.6 ± 11.9 (17–83)
<55	321 (74.8%)
≥55	108 (25.2%)
Male/female	94 (21.9%)/335 (78.1%)
Size of primary tumor (cm)	
Mean ± SD (range)	1.2 ± 0.9 (0.2–6.0)
<1.0	197 (45.9%)
≥1.0	232 (54.1%)
Multiplicity	
Single/Multiple	256 (59.7%)/173 (40.3%)
Bilaterality	
Absent/Present	311 (72.5%)/118 (27.5%)
ETE ^2^	
Absent/microscopic ETE/gross ETE	264 (61.5%)/69 (16.1%)/96 (22.4%)
T category *	
T1a	209 (48.7%)
T1b	97 (22.6%)
T2	24 (5.6%)
T3a	5 (1.2%)
T3b	75 (17.5%)
T4a	19 (4.4%)
N category *	
N0a	29 (6.7%)
N1a	304 (70.9%)
N1b	96 (22.4%)
Lymph node (LN) characteristics	
Number of removed LNs, median (range)	8 (3–102)
Number of metastatic LNs, median (range)	2 (0–41)
LN ratio, mean ± SD (range)	0.4 ± 0.3 (0.0–1.0)
Dose of administered ^131^I (GBq)	
3.70	63 (14.7%)
5.55	121 (28.2%)
6.66	245 (57.1%)
D0Tg ^3^ level (ng/mL)	
Mean ± SD (range)	8.1 ± 38.2 (0.0–500.0)
Interval between surgery and PET/CT (days)	
Mean ± SD (range)	79.7 ± 19.3 (31–185)

^1^ SD, standard deviation; ^2^ ETE, extra-thyroidal extension; ^3^ D0Tg, serum thyroglobulin level evaluated immediately before RAI therapy. * Staging according to the American Joint Committee on Cancer 8th Edition.

**Table 2:** Clinicopathological factors associated with the detection of FDG-avid persistent disease on PET/CT.

Parameters	FDG ^1^-Avid Persistent Disease (-)	FDG-Avid Persistent Disease (+)	Univariate Analysis	Multivariate Analysis	
	(*n* = 397)	(*n* = 32)	*p*	OR ^7^ (95% CI ^8^)	*p*
Age (year)			0.384		
<55	295 (91.9%)	26 (8.1%)			
≥55	102 (94.4%)	6 (5.6%)			
Sex			0.027	2.465 (0.913–6.658)	0.075
Female	315 (94.0%)	20 (6.0%)			
Male	82 (87.2%)	12 (12.8%)			
Size of tumor (cm)			0.001	2.350 (0.777–7.105)	0.131
<1.0	191 (96.9%)	6 (3.1%)			
≥1.0	206 (88.8%)	26 (11.2%)			
ETE ^2^			0.001	0.934 (0.024–36.808)	0.972
Absent or microscopic ETE	316 (94.9%)	17 (5.1%)			
Gross ETE	81 (84.4%)	15 (15.6%)			
Multiplicity			0.056		
Solitary	242 (94.5%)	14 (5.5%)			
Multiple	155 (89.6%)	18 (10.4%)			
Bilaterality			0.188		
Absent	291 (93.6%)	20 (6.4%)			
Present	106 (89.8%)	12 (10.2%)			
T category			<0.001	2.319 (0.057–93.616)	0.656
<3b	318 (94.9%)	17 (5.1%)			
≥3b	79 (84.0%)	15 (16.0%)			
N category			<0.001	2.039 (0.584–7.119)	0.262
N0a or N1a	319 (95.8%)	14 (4.2%)			
N1b	78 (81.2%)	18 (18.8%)			
Number of MLNs ^3^, Median (range)	2 (0–13)	6 (1–26)	<0.001	1.038 (0.057–1.128)	0.380
LNR ^4^, mean ± SD ^5^	0.38 ± 0.29	0.54 ± 0.29	0.004	4.074 (0.721–23.014)	0.111
D0Tg ^6^, mean ± SD	3.4 ± 10.6	66.0 ± 122.1	<0.001	1.043 (1.025–1.062)	<0.001

^1^ FDG, 2-deoxy-2-fluoro-D-glucose; ^2^ ETE, extra-thyroidal extension; ^3^ MLN, metastatic lymph node; ^4^ LNR, metastatic lymph node ratio (the number of metastatic lymph nodes/the number of dissected lymph nodes); ^5^ SD, standard deviation; ^6^ D0Tg, serum thyroglobulin level evaluated immediately before RAI therapy; ^7^ OR, odds ratio; ^8^ CI, confidence interval.

**Table 3:** Comparison of the diagnostic performance for the detection of FDG-avid persistent disease based on PET/CT indication identified using the suggested criteria.

Criteria	AUC ^4^	Sensitivity	Specificity	PPV ^5^	NPV ^6^	Accuracy	*p*-Value
LNR ^1^-combined	0.877	78.1	93.5	49.0	98.2	92.3	
D0Tg ^2^ > 6.0 (ng/mL)	0.876	84.4	89.2	38.6	98.6	88.8	<0.001 *
Number of MLN ^3^ > 5	0.649	53.1	81.1	18.5	95.6	79.0	<0.001 *
LNR > 0.51	0.574	59.4	74.6	15.8	95.8	73.4	<0.001 *

^1^ LNR, metastatic lymph node ratio; ^2^ D0Tg, serum thyroglobulin level evaluated immediately before RAI therapy; ^3^ MLN, metastatic lymph node; ^4^ AUC, area under the curve; ^5^ PPV, positive prediction value; ^6^ NPV, negative prediction value. * Each individual parameter was compared with the metastatic lymph nodes ratio-combined criteria.

## Data Availability

The datasets used and/or analyzed during the current study are available from the corresponding author on reasonable request.
